# Mussel-Inspired Adhesive Layer Supporting ZnO Nanorod Arrays Combined with Thiol-Ene Click Reaction for Constructing Multi-Level Carbon Fiber/Norbornene-Polyimide Interfaces

**DOI:** 10.3390/ma19050960

**Published:** 2026-03-02

**Authors:** Guoqiang Kong, Jianshun Feng, Meng Shao, Qiubing Yu, Zhenyu Liu, Kang Wang, Guang Yu, Xiang Zhao, Yan Huo, Xiaolei Guo, Qifen Wang, Zhe Sun, Haixiao Huang, Junwei Yu, Dayong Li, Bo Zhu

**Affiliations:** 1 Shandong Institute of Nonmetallic Materials, Jinan 250031, China; 2 Key Laboratory for Liquid Solid Structural Evolution and Processing of Materials, Ministry of Education, School of Materials Science and Engineering, Shandong University, Jinan 250061, China; 3 School of Electronic Engineering, Xidian University, Xi’an 710071, China; 4 Shandong University Institute for Advanced Studies in Education, Jinan 250100, China

**Keywords:** carbon fiber, PI-NA, C-PEI@OPDA, ZW, MPS, interfacial strength

## Abstract

Due to the non-polar and chemically inert nature of carbon fiber surfaces, the interfacial bonding strength between carbon fibers and norbornene-polyimide (PI-NA) resin matrix is relatively weak. To address this issue, this study constructed a composite coating on the carbon fiber surface and proposed a novel method to build robust interfaces based on multiple interfacial interactions, thereby effectively enhancing the interfacial properties between carbon fibers and PI-NA resin. Inspired by mussel adhesive proteins, this study established a multi-level synergistic interfacial reinforcement system by sequentially constructing a C-PEI@OPDA coating, in situ growing zinc oxide nanorods (ZW) arrays, and grafting 3-mercaptopropyltrimethoxysilane (MPS) onto carbon fiber surfaces. The C-PEI@OPDA coating, rich in amino (–NH_2_) and hydroxyl groups (–OH), enhanced adhesion to carbon fibers and adsorbed Zn^2+^ via coordination interactions to provide nucleation sites for ZW growth. Meanwhile, the active hydrogen in the coating promoted the crosslinking of PI-NA resin, thereby increasing the resin crosslinking density in the interfacial region. The vertically aligned ZW significantly increased surface roughness, enhanced mechanical interlocking effects, and provided secondary reaction sites for MPS grafting. The thiol groups (–SH) in MPS formed covalent bonds with PI-NA resin through thiol-ene click reactions, further strengthening interfacial bonding. The results showed that the ILSS, IFSS, and flexural strength of C-PEI@OPDA/ZW/MPS modified carbon fiber composites reached 75.15 MPa, 102.93 MPa, and 1735.56 MPa, representing improvements of 39.09%, 48.79%, and 31.16%, respectively. This study effectively enhanced the carbon fiber-reinforced polymer composites interfacial bonding strength through the synergistic effects of hydrogen bonding, mechanical interlocking, chemical bonding, and increased resin crosslinking density.

## 1. Introduction

Carbon fiber-reinforced polymer (CFRP) composites, owing to their high specific strength, high specific modulus, and excellent fatigue and corrosion resistance, have gradually expanded from cutting-edge aerospace applications to civilian industrial sectors such as automotive manufacturing, wind energy, and rail transportation, progressively replacing traditional metal components as the preferred materials for primary load-bearing structures [1,2,3,4,5]. However, the fiber/matrix interface, serving as the critical region for load transfer, directly determines the macroscopic mechanical response of composites [6,7,8]. When interfacial adhesion is poor, external loads cannot be effectively transferred to the reinforcing phase, causing premature interfacial debonding, interlaminar cracking, and other failure behaviors during service, which severely restricts the full realization of CFRP’s performance potential [9].

The fundamental cause of weak interfacial bonding lies in the inherent chemical inertness of the carbon fiber surface. The microstructure of carbon fiber is predominantly characterized by a turbostratic structure, with basal planes composed of hexagonal ring networks formed by sp^2^-hybridized carbon atoms through covalent bonds, where π electrons are highly delocalized [10,11]. This unique electronic configuration endows the carbon fiber surface with extremely low surface free energy and reactivity, exhibiting pronounced non-polarity and hydrophobicity. From a chemical perspective, the carbon fiber surface is virtually devoid of polar functional groups (such as hydroxyl (–OH), carboxyl (–COOH), and amino groups (–NH_2_)) that could serve as anchoring sites for resin molecules, making it difficult to establish effective chemical bonding or hydrogen bonding between fiber and matrix [12,13,14]. Furthermore, the smooth carbon fiber surface cannot provide sufficient capillary driving force for resin penetration, nor does it possess the microscale rough structures required for mechanical interlocking [15,16,17]. These interfacial defects at molecular and microscopic scales ultimately manifest macroscopically as low stress transfer efficiency and stress concentration in the interfacial region, subsequently inducing crack initiation and propagation. Therefore, developing effective surface modification strategies to enhance the surface activity and roughness of carbon fibers and to construct robust fiber/matrix interfacial bonding has become a core scientific challenge urgently requiring resolution in the field of composite materials.

To address the inherent chemical inertness of carbon fiber surfaces, researchers have developed various surface modification techniques to improve interfacial bonding with polymer matrices, primarily including oxidation treatment [18,19], plasma treatment [20], sizing treatment [21,22] and chemical grafting [23]. However, while these conventional modification methods improve interfacial properties, they are often accompanied by damage to the bulk mechanical properties of the fibers, and the interfacial enhancement effects remain limited. In recent years, nanomaterial modification techniques have attracted considerable attention due to their unique multi-scale interface regulation capabilities [24]. This approach involves the in situ growth or deposition of nanostructures on carbon fiber surfaces, such as carbon nanotubes [25,26], graphene oxide [27], and cerium oxide nanoparticles [28], which can construct micro-nanoscale rough morphologies on the fiber surface. These nanostructures significantly increase the specific surface area of the fibers, providing abundant physical interlocking sites for resin matrix penetration and anchoring, thereby enhancing the mechanical interlocking effect between fibers and matrix. However, during the process of composites bearing external loads, detachment of nanomaterials from carbon fiber surfaces constitutes one of the causes of interfacial failure [29].

To address this issue, polymer coatings with adhesive properties can be introduced onto carbon fiber surfaces, serving as growth templates to enhance the anchoring of nanomaterials [30]. Polydopamine (PDA) is a polymeric material formed by the self-polymerization of dopamine (DA) under alkaline conditions [31]. Its structure contains abundant–NH_2_ and–OH, enabling bonding with inorganic materials through hydrogen bonding, covalent bonding, and metal coordination interactions [32]. Polyethyleneimine (PEI) is a branched polymer rich in primary, secondary, and tertiary amine groups, possessing high reactivity and capable of undergoing Michael addition reactions and Schiff base reactions with DA under alkaline conditions to form chemically crosslinked networks [33]. Zinc oxide nanorods (ZW) possess abundant –OH on their surfaces and exhibit high aspect ratios, making them widely employed in interfacial modification studies of CFRP [34].

Therefore, this study utilizes the aforementioned reaction characteristics to improve the interfacial properties of carbon fiber-reinforced norbornene-terminated polyimide resin composites (CF/PI). A C-PEI@OPDA coating was constructed on the carbon fiber surface through the copolymerization of PEI and DA under alkaline conditions. This coating contains abundant active hydrogen, which can promote the curing reactions between olefinic groups in the norbornene-terminated polyimide resin (PI-NA) molecular chains, thereby increasing the resin crosslinking density in the interfacial region. Subsequently, using the C-PEI@OPDA coating as a growth template and exploiting the capability of this polymer coating to adsorb metal ions and undergo coordination interactions, ZW were grown in situ on its surface to increase the carbon fiber surface roughness, thereby facilitating mechanical interlocking between carbon fibers and PI resin. In previous studies by our research group, it has been demonstrated that the thiol groups (–SH) in 3-mercaptopropyltrimethoxysilane (MPS) can form covalent bonds with the norbornene groups in PI through thiol-ene click reactions [35]. Therefore, on this basis, MPS was further grafted onto the fiber surface to introduce covalent bonding at the carbon fiber/resin interface. Through the synergistic effects of multiple mechanisms including hydrogen bonding, mechanical interlocking, and chemical bonding, this work aims to enhance the interfacial bonding strength between carbon fibers and PI-NA resin, thereby improving the interfacial properties of the composites. In this study, a systematic analysis was conducted on C-PEI@OPDA/ZW/MPS modified carbon fibers and their composites using multiple characterization techniques. Ultraviolet-visible spectroscopy (UV-Vis) was employed to analyze the evolution of functional groups in DA and DA/PEI solutions during the reaction process. Thermogravimetric analysis (TGA) was used to evaluate the thermal stability of the C-PEI@OPDA coating. Scanning electron microscopy (SEM) was utilized to observe the surface morphology changes of carbon fibers before and after modification. X-ray photoelectron spectroscopy (XPS) was applied to analyze the evolution of surface chemical composition during the stepwise modification process. X-ray diffraction (XRD) was performed to characterize the phase composition on the carbon fiber surface. Contact angle and surface energy measurements were conducted to investigate the variation in surface polarity during the modification process. Furthermore, the improvement in interfacial properties of CF/PI was evaluated through mechanical property tests including interlaminar shear strength (ILSS), interfacial shear strength (IFSS), and flexural strength. Finally, based on the analysis of interfacial microstructure, the synergistic mechanism by which the C-PEI@OPDA coating, ZW, and MPS grafting enhance the mechanical properties of carbon fiber composites was elucidated.

## 2. Experiment Preparation

### 2.1. Pre-Treatment of Carbon Fiber

Carbon fiber bundles were placed in a muffle furnace and heat-treated at 380 °C for 2 h under air atmosphere to remove the surface sizing agent, and the resulting samples were designated as De-CF. Subsequently, the desized carbon fibers were immersed in a 30 wt% H_2_O_2_ solution and subjected to oxidation treatment at 60 °C for 2 h. This oxidation treatment was intended to enrich the carbon fiber surface with oxygen-containing functional groups such as –OH and –COOH groups, thereby enhancing the surface reactivity and providing active sites for subsequent grafting of the C-PEI@OPDA coating. The oxidized carbon fibers were designated as CFO.

### 2.2. Preparation Process of C-PEI@OPDA/ZW/MPS Composite Coating on Carbon Fiber Surface

(1) Preparation of precursor solutions for carbon fiber modification

Zinc acetate dihydrate (1 mmol) was dissolved in 80 mL of ethanol, heated to 50 °C, and stirred for 30 min before cooling to room temperature. Subsequently, 40 mL of the above solution was taken and diluted with 320 mL of ethanol to obtain a diluted zinc acetate dihydrate solution. A sodium hydroxide solution with a concentration of 2 mM was prepared using 100 mL of ethanol as the solvent. Then, 40 mL of this sodium hydroxide solution was taken and diluted with 100 mL of ethanol to obtain a diluted sodium hydroxide solution. The diluted zinc acetate dihydrate solution and sodium hydroxide solution were mixed and brought to a final volume of 800 mL with ethanol. The mixture was heated to 65 °C, stirred for 30 min, and then allowed to stand for 1 h to obtain a transparent “seed” solution. Zinc nitrate hexahydrate (3.71 g) and hexamethylenetetramine (1.75 g) were dissolved in 300 mL of deionized water and stirred for 60 min to obtain the growth solution.

(2) Deposition of C-PEI@OPDA or OPDA on carbon fiber surface

A tris(hydroxymethyl)aminomethane (Tris) aqueous solution (500 mL, 10 mM) was prepared, and its pH was adjusted to 8.5 using 1 M hydrochloric acid to obtain the buffer solution. Subsequently, CFO along with PEI (2 mg/mL) and DA (2 mg/mL) at a mass ratio of 1:1 were added into the buffer solution. The mixture was then heated to 70 °C and subjected to ultrasonic treatment at 60 kHz for 3 h. After modification, the samples were removed, washed five times with deionized water, and dried in a vacuum oven at 60 °C for 12 h. The resulting carbon fibers were designated as CF@E-OP. For comparison, a control sample was prepared following the same procedure but without PEI addition, using only DA (2 mg/mL) for modification. The resulting fibers were designated as CF@OP.

(3) Growth of ZW on CF@E-OP surface

CFO and CF@E-OP were immersed in the “seed” solution for 5 min, followed by drying in an oven for 10 min. This process was repeated five times to ensure sufficient adsorption of the “seeds” onto the carbon fiber surface. The seed-loaded CFO and CF@E-OP were then immersed in 300 mL of the growth solution. The growth solution together with the carbon fibers was transferred into a polytetrafluoroethylene (PTFE)-lined autoclave for hydrothermal reaction at 90 °C for 10 h. After the autoclave cooled to room temperature, the carbon fibers were removed and washed alternately with ethanol and deionized water five times. Subsequently, the samples were dried in a vacuum oven at 60 °C for 12 h. The resulting samples were designated as CF@ZW and CF@E-OP/ZW, respectively. The relevant parameters are listed in Table 1.

(4) Grafting of MPS onto CF@E-OP/ZW

A mixed solvent was prepared by combining 250 mL of deionized water and 250 mL of ethanol (volume ratio 1:1). The pH of the mixture was then adjusted to 5 using acetic acid, and a predetermined amount of MPS was added. The solution was stirred at room temperature for 6 h to obtain a 0.5 wt% MPS hydrolysis solution. CF@E-OP/ZW samples were immersed in the MPS hydrolysis solution and treated at 70 °C for 2 h. After treatment, the samples were washed three times with anhydrous ethanol and subsequently dried at 120 °C for 2 h to achieve chemical grafting of MPS onto the carbon fiber surface. The relevant parameters are listed in Table 2. The resulting samples were designated as CF@E-OP/ZWS. The nomenclature of each car bon fiber sample, along with the corresponding modification steps and their purposes, are detailed in Table 3. The details regarding the equipment, sample preparation and conditions for the characterization of samples are presented in the manuscript-Supplementary Materials.

## 3. Results

**Figure 1:**
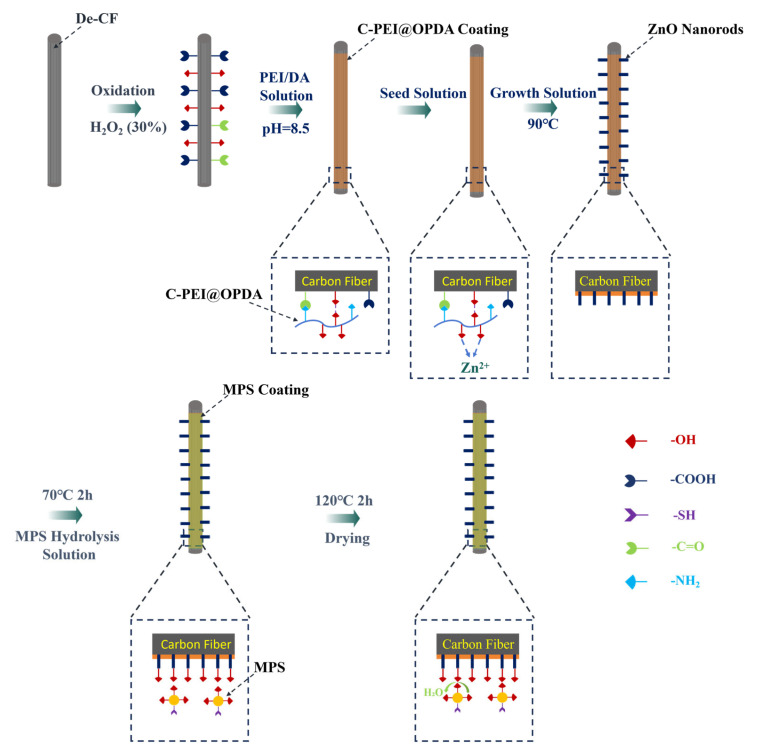
Schematic diagram of the growth of C-PEI@OPDA/ZW/MPS composite coating on the surface of carbon fiber.

The preparation process of the C-PEI@OPDA/ZW/MPS composite coating on carbon fiber surface is illustrated in Figure 1. As shown in the figure, after hydrogen peroxide oxidation treatment, oxygen-containing functional groups such as –COOH, –OH, and C=O were introduced onto the carbon fiber surface, providing active sites for subsequent physical adsorption and chemical bonding. The oxidized carbon fibers were then immersed in the PEI/DA solution. Under alkaline conditions, the –NH_2_ groups in PEI reacted with the quinone groups and α,β-unsaturated structures formed during DA oxidation through Schiff base reactions and Michael addition reactions to form chemical bonds, followed by self-polymerization to form C-PEI@OPDA. Simultaneously, the –NH_2_ groups in C-PEI@OPDA underwent Schiff base reactions with the C=O groups on the oxidized carbon fiber surface to form chemical bonds. Additionally, hydrogen bonds were formed between the polar groups in C-PEI@OPDA and the oxygen-containing groups on the carbon fiber surface. Based on these multiple interactions, the coating firmly adhered to the carbon fiber surface. Subsequently, the carbon fibers coated with C-PEI@OPDA were immersed in a seed solution, during which Zn^2+^ ions formed coordination interactions with the phenolic –OH in C-PEI@OPDA and were adsorbed onto the carbon fiber surface [36]. The seeded carbon fibers were then immersed in a growth solution, where the ZnO seeds grew preferentially along the c-axis direction, ultimately forming vertically aligned ZW on the carbon fiber surface. MPS underwent hydrolysis reactions in an acidic water/ethanol mixed solution, during which the three methoxy groups in its molecular structure were hydrolyzed to form Si–OH groups. The ZW surface contains –OH groups originating from lattice oxygen defects and the dissociation of adsorbed water molecules. Therefore, using ZW as secondary reaction sites, MPS was grafted onto the carbon fiber surface. During the grafting process, the Si–OH groups in MPS underwent dehydration condensation reactions with the –OH groups on the ZW surface at 120 °C to form Si–O–Zn bonds, while adjacent MPS molecules formed siloxane network structures through polycondensation reactions, thereby creating an organosilane coating on the ZW surface and in the interstices. The –SH groups on the outer surface of the MPS coating serve as reactive functional groups that can participate in the crosslinking and curing reactions of the PI resin matrix during subsequent composite molding. Based on the synergistic effects of these multi-scale and multi-mechanism interactions, CF/PI with excellent interfacial enhancement effects were ultimately prepared.

**Figure 2:**
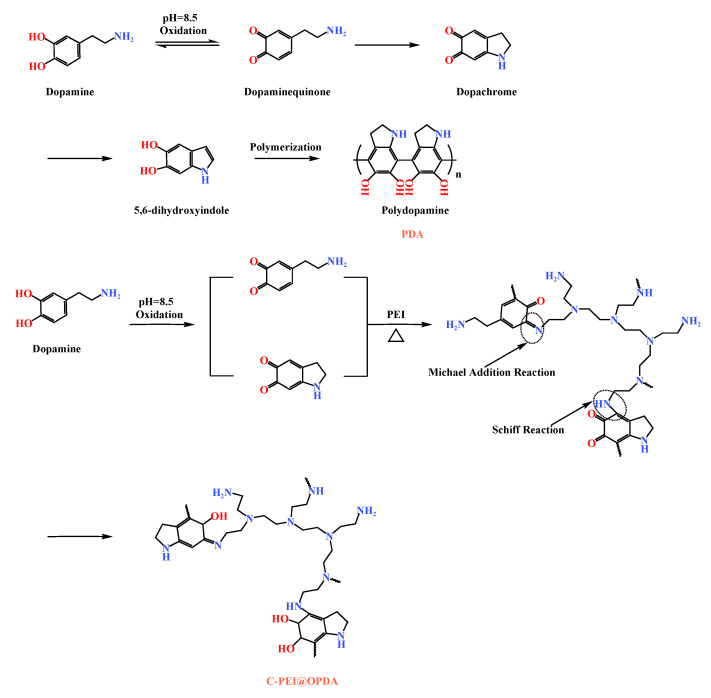
The formation mechanisms of PDA and C-PEI@OPDA.

Figure 2 illustrates the formation mechanisms of PDA and C-PEI@OPDA. As shown in the figure, in a weakly alkaline dopamine-Tris solution (pH = 8.5), the catechol groups in dopamine were oxidized to dopamine-quinone structures under the promotion of ultrasonic cavitation. Subsequently, the dopamine-quinone structures underwent nucleophilic reactions and rearrangement to form the intermediate 5,6-dihydroxyindole (DHI) [37]. These intermediates were further crosslinked through radical polymerization or Michael addition reactions to form polydopamine. PEI is a polymer rich in primary amine groups, and this characteristic can be utilized to modify PDA. Under alkaline conditions, the primary amine groups in PEI reacted with the carbonyl groups of the intermediates formed during dopamine polymerization through Schiff base reactions and Michael addition reactions to form C=N and C–N bonds, respectively, thereby generating C-PEI@OPDA [38].

**Figure 3:**
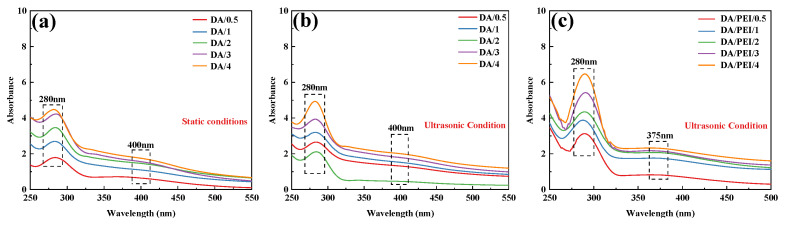
UV-Vis absorption spectra: (**a**) DA solution under static conditions, (**b**) DA solution under ultrasonic treatment, and (**c**) DA/PEI solution under ultrasonic treatment.

The absorbance changes in DA and DA/PEI solutions under different conditions over time were analyzed by UV-Vis spectroscopy. As shown in Figure 3a,b, an absorption peak appeared at approximately 280 nm, which corresponds to quinone compounds (such as dopamine-quinone), and the intensity of this peak increased significantly with prolonged reaction time. Under ultrasonic treatment, the cavitation effect generates a large amount of reactive oxygen species (ROS). In the presence of ROS, the catechol groups lose electrons and are oxidized to quinone structures. Consequently, the absorption peak intensity of quinone compounds in the ultrasonically treated DA solution was generally higher than that in the untreated DA solution. Additionally, a weak broad absorption peak was observed at approximately 400 nm, which is associated with the highly conjugated macromolecular structure of PDA. As shown in Figure 3c, in the DA/PEI solution, the broad absorption peak at 400 nm was replaced by a new absorption peak at 365 nm. This new peak is attributed to the n→π* transition of the C=C–C=N structure [39]. Furthermore, with increasing ultrasonic treatment time, the intensity of the absorption peak at 365 nm increased, indicating that the cavitation effect produced more quinone compounds, which facilitated more Schiff base reactions and Michael addition reactions between DA and PEI. As a result, the concentration of C=C–C=N structures in the solution increased over time.

**Figure 4:**
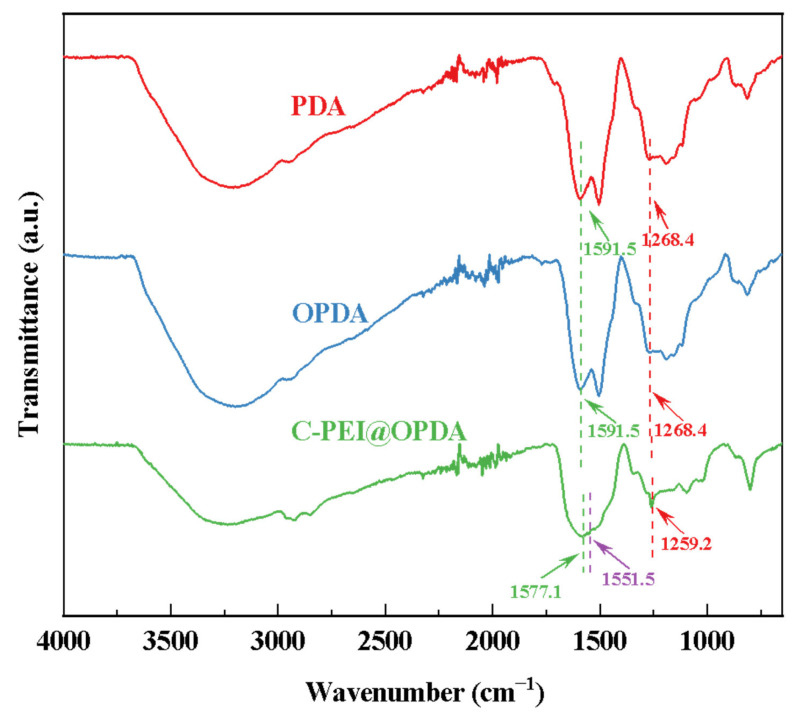
The infrared spectra of PDA, OPDA, and C-PEI@OPDA.

Figure 4 shows the FTIR spectra of PDA prepared from DA solution and C-PEI@OPDA polymer prepared from DA/PEI solution. Without ultrasonic treatment, the PDA deposited from DA exhibited two characteristic peaks at 1268.4 cm^−1^ and 1591.5 cm^−1^, corresponding to the C–OH stretching vibration and N–H bending vibration, respectively [40]. The characteristic peak positions of OPDA prepared under ultrasonic conditions were consistent with those of PDA, indicating that ultrasonic cavitation did not introduce new functional groups. For C-PEI@OPDA, the peaks corresponding to C–OH and N–H were located at 1259.2 cm^−1^ and 1577.1 cm^−1^, respectively, both of which shifted to lower wavenumbers compared to PDA and OPDA. This red-shift phenomenon can be attributed to the fact that the –NH_2_ in the introduced PEI altered the electron density distribution of C–OH and N–H through hydrogen bonding interactions, thereby causing the peak positions to shift. Moreover, a new characteristic peak appeared at 1551.5 cm^−1^ for C-PEI@OPDA, which corresponds to the stretching vibration of the C=N bond [41]. This result confirms that a copolymerization reaction occurred between PEI and DA.

**Figure 5:**
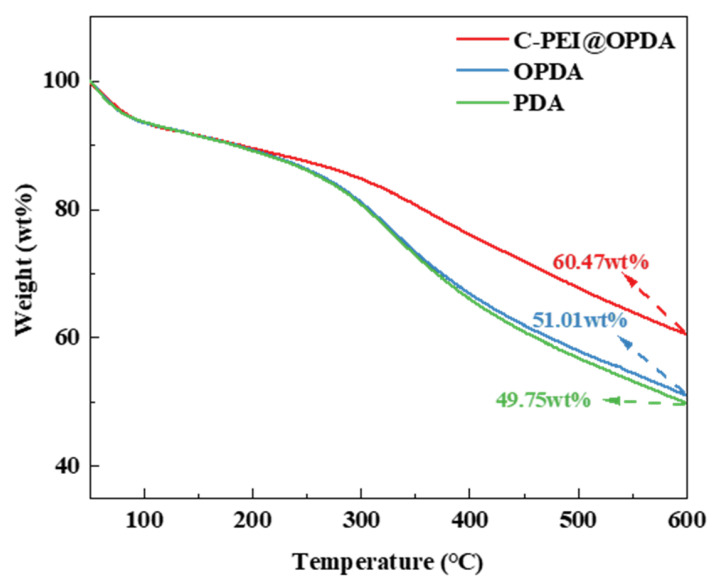
TG of PDA, OPDA, and C-PEI@OPDA.

Since the subsequent composite preparation process needs to be conducted under high-temperature conditions, the thermal stability of the polymer coatings was first evaluated by thermogravimetric analysis (TGA). Figure 5 shows the thermal stability curves of PDA, OPDA, and C-PEI@OPDA in the temperature range of 50 °C to 600 °C. The results indicate that at 600 °C, the mass residual rates of PDA, OPDA, and C-PEI@OPDA were 49.75 wt%, 51.01 wt%, and 60.47 wt%, respectively. All samples exhibited varying degrees of mass loss, which can be attributed to the desorption of water molecules and the decomposition of aromatic rings, –OH, –COOH, and C=O groups in the polymer backbone at elevated temperatures. The mass residual rate of OPDA was slightly higher than that of PDA, which may be because the ultrasonic treatment process increased the degree of polymerization of OPDA, thereby improving its thermal resistance. PEI is a branched polymer containing abundant –NH_2_ groups, and the primary amines in its molecular chains possess high reactivity. Compared with PDA and OPDA, C-PEI@OPDA exhibited superior thermal stability because the primary amines in PEI can react with the double bond intermediates and quinone groups generated during the DA oxidation process to form chemical bonds, thereby increasing the crosslinking density of C-PEI@OPDA. The highly crosslinked structure restricts the movement of C-PEI@OPDA molecular chain segments, which means that higher energy is required to destroy this crosslinked structure. Therefore, the thermal stability of C-PEI@OPDA was significantly enhanced.

**Figure 6:**
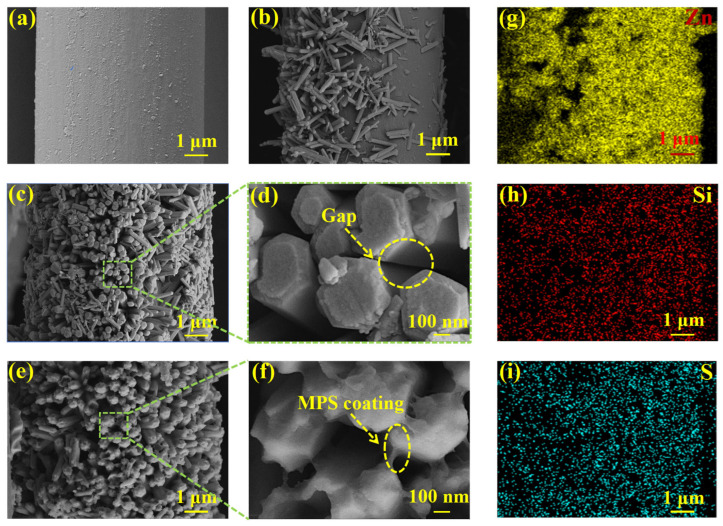
SEM images: (**a**) CF@E-OP, (**b**) CF@ZW, (**c**) CF@E-OP/ZW, (**d**) partial CF@E-OP/ZW, (**e**) CF@E-OP/ZWS, (**f**) partial CF@E-OP/ZWS, and (**g**–**i**) low-magnification CF@E-OP/ZWS EDS elemental surface distribution results.

As shown in Figure 6a, after the deposition of the C-PEI@OPDA coating, a distinct polymer coating was formed on the CF@E-OP surface, which provided abundant active sites for ZW growth. As shown in Figure 6b, ZW grown on the carbon fiber surface without C-PEI@OPDA coating modification exhibited sparse distribution and disordered arrangement. However, when ZW was grown on the carbon fiber surface using the C-PEI@OPDA coating as a growth template, as shown in Figure 6c,d, nanorods were observed to be regularly and vertically aligned on the carbon fiber surface with gaps between the rods. The reason for the above morphological differences lies in the fact that the C-PEI@OPDA coating contains abundant polar groups such as –OH and –NH_2_, which can adsorb Zn^2+^ and promote uniform nucleation, thereby inducing preferential growth of ZW in the vertical direction. In contrast, without the C-PEI@OPDA coating as a growth template, the surface chemical properties of the carbon fiber were unfavorable for regular ZnO nucleation, resulting in disordered ZW growth. As shown in Figure 6e,f, the MPS coating was observed on the ZW surface and in the interstices of CF@E-OP/ZWS. The corresponding elemental mapping results are shown in Figure 6g–i, and the results of CF@E-OP/ZWS showed distinct distribution signals of Zn, Si, and S elements, which further confirmed that ZW and MPS were successfully loaded onto the carbon fiber surface.

**Figure 7:**
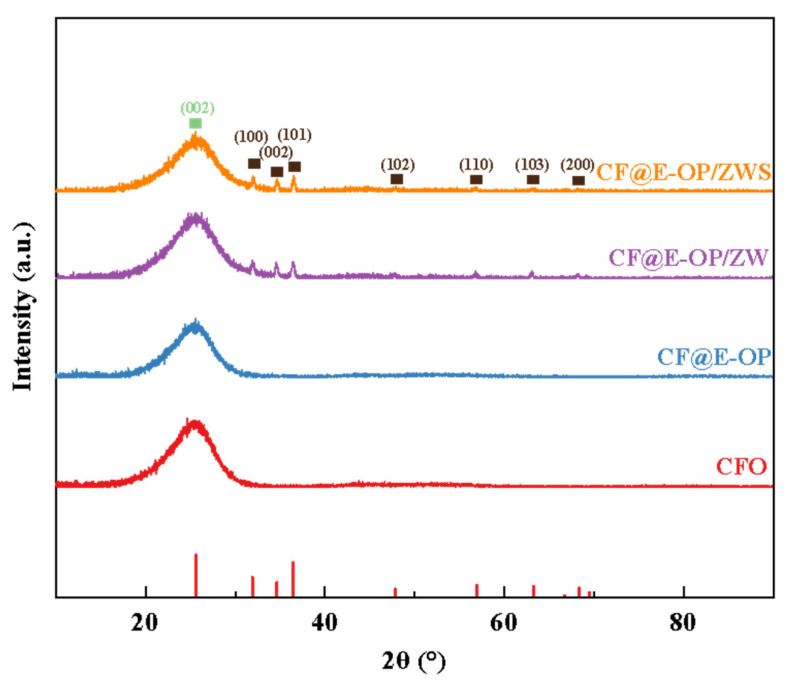
XRD patterns of CFO, CF@E-OP, CF@ZW, CF@E-OP/ZW and CF@E-OP/ZWS.

As shown in Figure 7, all samples exhibited a sharp diffraction peak at 2θ = 25.5°, which corresponds to the (002) crystal plane of the graphitized structure on the carbon fiber surface. For CF@ZW, a series of weak diffraction peaks were observed at 2θ = 31.9°, 34.6°, 36.4°, 47.8°, 56.9°, 63.2°, and 66.7°, which correspond to the (100), (002), (101), (102), (110), (103), and (200) crystal planes of the ZnO hexagonal wurtzite phase, respectively, indicating that ZW was deposited on the carbon fiber surface [42,43]. Compared with CF@ZW, the intensity of the ZnO diffraction peaks in CF@E-OP/ZW increased, indicating that using the C-PEI@OPDA coating as a growth template can promote ZW growth on the carbon fiber surface, thereby increasing the loading amount of ZW on the fiber surface. After further grafting MPS onto the CF@E-OP/ZW surface to obtain CF@E-OP/ZWS, the characteristic diffraction peak intensity of ZnO on the carbon fiber surface did not change significantly compared with CF@E-OP/ZW, indicating that the MPS grafting process does not affect the crystal structure of ZW on the carbon fiber surface.

**Figure 8:**
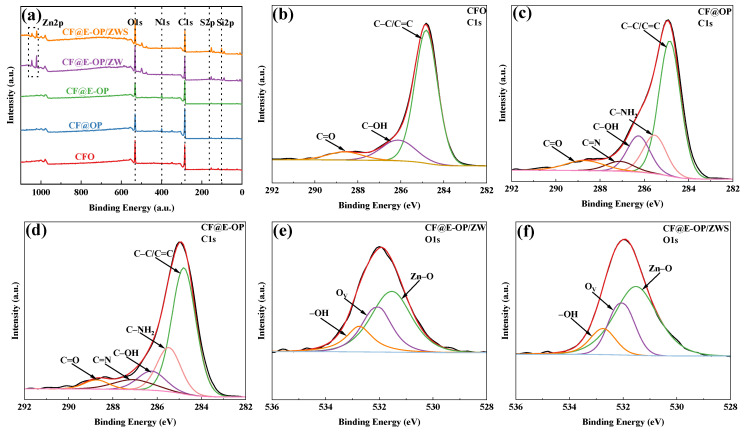
(**a**) XPS survey spectra of CFO, CF@OP, CF@E-OP, CF@E-OP/ZW, and CF@E-OP/ZWS, (**b**) CFO, (**c**) CF@OP, (**d**) CF@E-OP C1s peak-fitted XPS spectra, (**e**) CF@E-OP/ZW, and (**f**) CF@E-OP/ZWS O1s peak-fitted XPS spectra.

As shown in the XPS survey spectra in Figure 8a, all samples were predominantly composed of C and O elements. A small amount of N element was detected in CF@OP and CF@E-OP. CF@E-OP/ZW contained small amounts of N and Zn elements, while CF@E-OP/ZWS contained small amounts of N, Zn, Si, and S elements. Elemental analysis reveals that the detection of N elements confirms the successful coating of C-PEI@OPDA on the carbon fiber surface. The appearance of Zn elements indicates the effective growth of ZW, while the presence of S elements and Si elements further verifies the successful grafting of MPS. The elemental composition analysis of each sample is presented in Table 4. Compared with CFO, the N element content in CF@OP was 5.95%, which is related to the deposition of –NH_2_-containing OPDA on the carbon fiber surface. The N element content in CF@E-OP increased to 7.50% because the branched structure of PEI provided more –NH_2_ for the C-PEI@OPDA coating, thereby increasing the N element content on the carbon fiber surface. The detection of 2.69% Zn element in CF@E-OP/ZW is related to the growth of ZW on the carbon fiber surface. The contents of Si and S in CF@E-OP/ZWS were 11.76% and 1.13%, respectively, indicating that MPS containing Si and S elements was grafted onto the carbon fiber surface. As shown in Figure 8b–d, peak fitting was performed on the C1s spectra of CFO, CF@OP, and CF@E-OP. The C1s spectrum of CFO can be fitted into three component peaks, located at 284.8 eV for C–C/C=C, 286.2 eV for C–OH, and 288.7 eV for C=O [44]. Compared with CFO, the peak fitting results of CF@OP and CF@E-OP showed that, in addition to the above components, two additional component peaks were fitted, namely C–NH_2_ at 285.5 eV and C=N at 287.1 eV [45]. Table 5 further illustrates the variation patterns of chemical components on the carbon fiber surface during the modification process. According to the data in the table, the surface components of CFO included not only the dominant C–C/C=C (75.81%) but also 17.39% C–OH and 6.8% C=O generated by oxidative etching. Analysis of the surface functional group content of CF@OP indicated that 16.67% C–NH_2_ appeared on its surface, which is related to the loading of NH_2_-rich OPDA on the fiber surface. Additionally, 5.00% C=N appeared in this sample, which can be attributed to the formation of C=N bonds through Schiff base reactions between DA monomers and between NH_2_ in OPDA and C=O on the carbon fiber surface. After modifying the carbon fiber with C-PEI@OPDA, the C–NH_2_ content on the CF@E-OP surface increased to 20.23% compared with CF@OP, which can be attributed to the abundant NH_2_ groups in the PEI backbone of C-PEI@OPDA. Meanwhile, the C–OH content decreased to 10.40%, which is attributed to the Michael addition reaction between NH_2_ in PEI and the o-quinone formed by DA oxidation, where the o-quinone originates from the oxidation of phenolic –OH, resulting in the consumption of this functional group. Furthermore, the C=N content increased to 7.51% compared with CF@OP, while C=O decreased to 4.06%. This result indicates that the abundant NH_2_ in CF@E-OP causes more consumption of C=O through Schiff base reactions, thereby generating more C=N. As shown in Figure 8e,f, the O1s XPS spectra of CF@E-OP/ZW and CF@E-OP/ZWS were both fitted with one main peak and two secondary peaks at higher binding energies. The main peak at lower binding energy (531.5 eV) is associated with Zn–O–Zn in lattice oxygen [46,47]. The peak at medium binding energy (532.1 eV) corresponds to Zn–OH coordinated with Zn^2+^ cations in the oxygen-deficient regions on the ZW surface [48]. The peak at higher binding energy (532.7 eV) corresponds to –OH groups adsorbed on the ZW surface. Table 5 compares the changes in oxygen element chemical states between CF@E-OP/ZW and CF@E-OP/ZWS after MPS grafting treatment. The results showed that the contents of Zn–O–Zn, Zn–OH, and –OH in CF@E-OP/ZW were 52.63%, 30.00%, and 17.37%, respectively. After MPS grafting treatment, the contents of Zn–OH and –OH in CF@E-OP/ZWS decreased to 26.20% and 14.28%, respectively. This change can be attributed to the formation of chemical bonds between the –OH adsorbed on the ZW surface and Zn–OH with Si–OH in MPS through dehydration condensation reactions.

**Figure 9:**
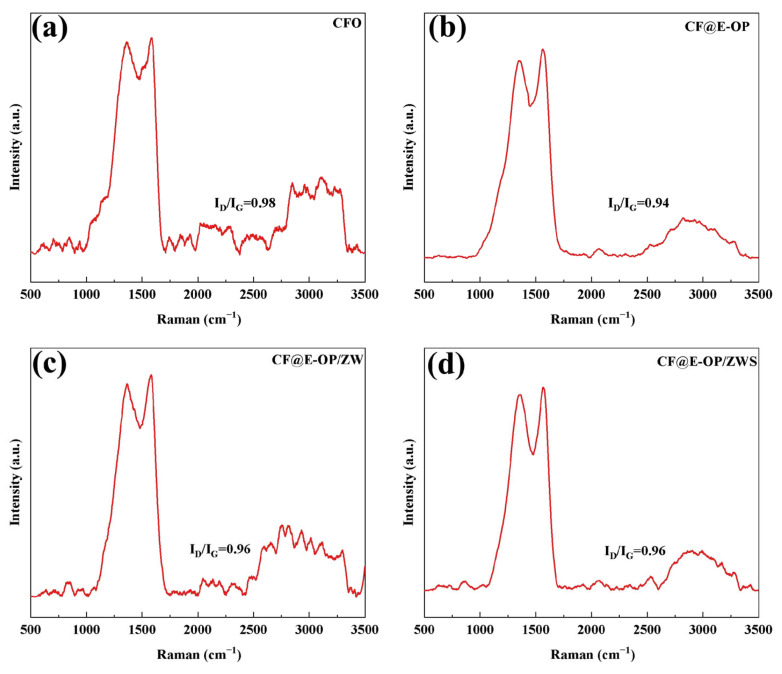
Raman spectra of (**a**) CFO, (**b**) CF@E-OP, (**c**) CF@E-OP/ZW, and (**d**) CF@E-OP/ZWS.

Raman spectroscopy can be used to analyze the surface defect characteristics and changes in ordered structure of modified carbon fibers. Each spectrum contains two characteristic bands: the D band at 1360 cm^−1^ and the G band at 1580 cm^−1^ [49]. The D band is associated with the amorphous structure or defects of carbon, while the G band originates from the ordered graphitized structure of carbon. The peak intensity ratio of the D band to the G band is used to evaluate the degree of graphitization on the carbon fiber surface. As shown in Figure 9a, the I_D_/I_G_ of CFO was 0.98. After C-PEI@OPDA deposition, the G peak of the CF@E-OP sample was enhanced, and its I_D_/I_G_ value decreased to 0.94 (as shown in Figure 9b). This change can be attributed to two aspects: first, the highly crosslinked structure of C-PEI@OPDA reduced the degree of disorder on the carbon fiber surface; second, the π-orbital hybridization between the aromatic ring structures in C-PEI@OPDA and the graphite structure on the carbon fiber surface enhanced the local order of the fiber surface. As shown in Figure 9c, compared with CF@E-OP, the I_D_/I_G_ of the CF@E-OP/ZW sample increased to 0.96. ZW itself does not affect the graphite structure on the carbon fiber surface. The increase in this ratio may be due to the fact that during the hydrothermal growth of ZW, the crosslinked network structure in the C-PEI@OPDA coating on the carbon fiber surface was damaged to a certain extent, resulting in an increase in the degree of disorder on the carbon fiber surface. As shown in Figure 9d, compared with CF@E-OP/ZW, the I_D_/I_G_ of CF@E-OP/ZWS showed no change, indicating that the silane MPS grafting treatment does not affect the graphitized structure on the carbon fiber surface.

**Figure 10:**
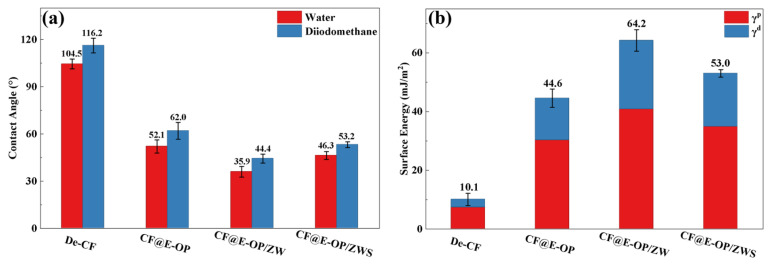
(**a**) Contact angles of carbon fibers with water and diiodomethane before and after modification and (**b**) surface energy of fibers before and after modification.

The water contact angles of carbon fiber samples before and after modification were measured, and the changes in their surface energy were further analyzed to investigate the effects of C-PEI@OPDA coating deposition, ZW growth, and MPS grafting treatment on the surface affinity of carbon fibers. As shown in Figure 10a, the unmodified carbon fiber surface exhibited chemical inertness, which made it difficult for water droplets to spread on its surface, resulting in a high water contact angle of 104.5°. After chemical deposition of the C-PEI@OPDA coating, the water contact angle of the carbon fiber decreased to 52.1° because abundant polar groups such as –NH_2_, –OH, and C=O were introduced onto the CF@E-OP surface, thereby increasing the interaction between the carbon fiber surface and water droplets. When ZW was further grown on the carbon fiber surface using the C-PEI@OPDA coating as a growth template, the water contact angle decreased to 35.9°. This can be attributed to the fact that the numerous gaps between the vertically grown ZW on the carbon fiber surface formed a capillary channel network. When water droplets contacted the carbon fiber surface, they spread on the fiber surface through capillary penetration effects. Meanwhile, ZW itself possesses abundant –OH groups that can form hydrogen bonds with water molecules, thereby enhancing the capillary penetration effect. Compared with CF@E-OP/ZW, the water contact angle of sample CF@E-OP/ZWS after MPS grafting increased to 46.3°. Although MPS contains polar –SH groups, the polycondensation reaction between Si–OH and –OH on the ZW surface caused a reduction in the content of polar functional groups on the carbon fiber surface, thereby decreasing the density of interaction sites between the fiber and water droplets. As shown in Figure 10b, the surface energy of De-CF was 10.1 mJ/m^2^, mainly because the desized carbon fiber surface was smooth and contained few oxygen-containing groups, resulting in low surface energy. After chemical deposition of the C-PEI@OPDA coating, the surface energy of CF@E-OP increased to 44.6 mJ/m^2^. This is mainly attributed to the fact that the introduced polar functional groups such as –NH_2_ and –OH increased the polar component of the fiber surface. For CF@E-OP/ZW, the growth of ZW further increased the number of polar functional groups on the carbon fiber surface, thereby increasing the surface energy to 64.2 mJ/m^2^. For CF@E-OP/ZWS, the MPS grafting reaction consumed part of the –OH on the ZW surface, thus further reducing the surface energy to 53.0 mJ/m^2^.

**Figure 11:**
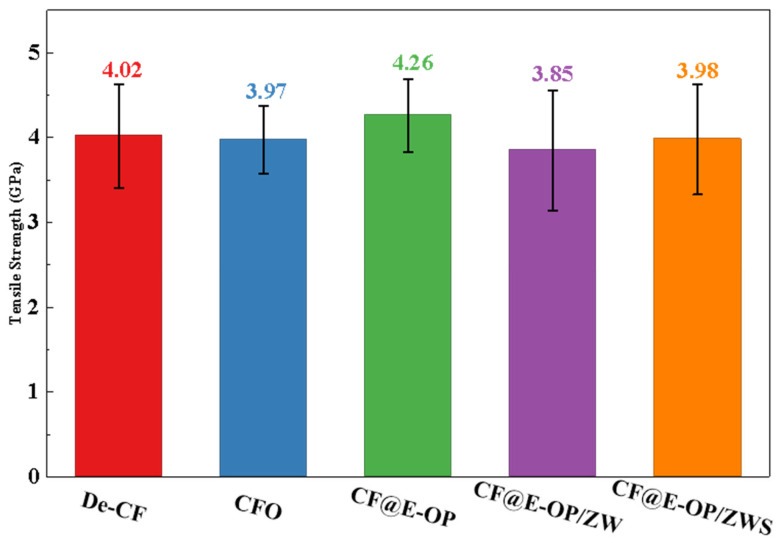
Single filament tensile strength of De-CF, CFO, CF@E-OP, CF@E-OP/ZW and CF@E-OP/ZWS.

To evaluate the changes in mechanical properties of carbon fibers during the stepwise modification process with C-PEI@OPDA coating, ZW, and MPS, single fiber tensile strength tests were conducted on the modified samples. As shown in Figure 11, the tensile strength of desized carbon fiber was 4.02 GPa. After deposition of the C-PEI@OPDA coating, the tensile strength of CF@E-OP increased to 4.26 GPa. This improvement can be attributed to the fact that the C-PEI@OPDA coating formed by in situ polymerization adhered tightly to the carbon fiber surface, thereby repairing the micro-defects on the carbon fiber surface to a certain extent and improving the mechanical properties of the single carbon fiber. After growing ZW on the carbon fiber surface by hydrothermal method, the single fiber tensile strength of CF@E-OP/ZW decreased to 3.85 GPa. This may be because the extreme high-pressure conditions during the hydrothermal reaction and the alkaline growth environment caused certain damage to both the graphite microcrystals in the carbon fiber bulk and the C-PEI@OPDA coating on its surface. Meanwhile, the ZW constructed protruding structures on the carbon fiber surface, which may become stress concentration points during the tensile process, making the single fiber more susceptible to fracture under stress. After further treatment with MPS grafting, compared with CF@E-OP/ZW, the single fiber tensile strength of CF@E-OP/ZWS slightly increased to 3.98 GPa. This may be attributed to the fact that MPS penetrated through the gaps of the ZW to the carbon fiber surface and formed a silane coating through polycondensation, thereby providing a certain degree of reinforcement to the mechanical strength of the carbon fiber.

**Figure 12:**
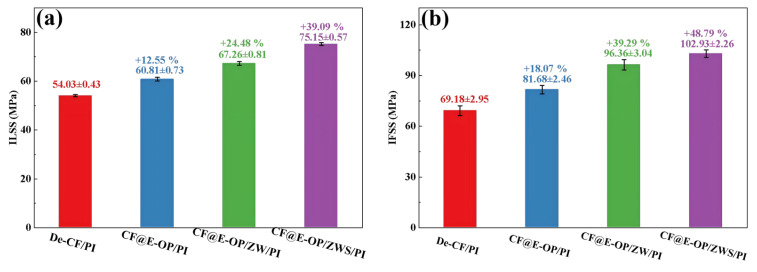
(**a**) The ILSS and (**b**) IFSS of CF/PI, each group was tested five times, and data are presented as mean ± SD (n = 5).

ILSS and IFSS were used to evaluate the effects of C-PEI@OPDA coating, ZW, and MPS coating on the CF/PI interfacial strength. As shown in Figure 12a, compared with the composites prepared from desized carbon fibers, the ILSS of CF@E-OP/PI prepared from C-PEI@OPDA modified carbon fibers increased by 12.55%. This improvement is related to the abundant polar functional groups such as –OH and –NH_2_ in C-PEI@OPDA. According to the wetting-adsorption theory, the greater the surface polarity of the carbon fiber, the easier it is for the liquid PI resin to spread on the fiber surface. This not only increases the contact area between the fiber and resin but also enhances the hydrogen bond density between –OH and –NH_2_ on the carbon fiber surface and C=O in the imide rings of PI molecular chains. In addition, the active hydrogen atoms in –NH_2_ can promote the crosslinking reaction of norbornene end groups in PI molecular chains, thereby increasing the crosslinking density of the resin in the interfacial region. Under the synergistic effect of the above factors, the interfacial adhesion between carbon fiber and PI resin was improved. When ZW was grown on the carbon fiber surface by hydrothermal method using the C-PEI@OPDA coating as a growth template, the ILSS of the prepared CF@E-OP/ZW/PI increased by 24.48% compared with De-CF/PI. This result can be attributed to the fact that ZW increased the surface roughness of the carbon fiber, thereby enhancing the mechanical interlocking effect between the carbon fiber and PI resin. Meanwhile, the PI resin that penetrated into the ZW gaps can form hydrogen bonds with –OH and –NH_2_ in the C-PEI@OPDA coating. Therefore, based on the synergistic effect of physical interlocking and hydrogen bonding, the ILSS value was further improved. On this basis, after further grafting MPS onto the ZW surface, the ILSS of the prepared CF@E-OP/ZWS/PI increased by 39.09% compared with De-CF/PI. This is mainly because the –SH in MPS can form chemical bonds with the norbornene end groups in PI, thereby strengthening the interfacial adhesion between carbon fiber and PI resin. As shown in Figure 12b, compared with De-CF/PI, the IFSS values of CF@E-OP/PI, CF@E-OP/ZW/PI, and CF@E-OP/ZWS/PI increased by 18.07%, 39.29%, and 48.79%, respectively, which is consistent with the trend of ILSS changes. When carbon fibers were treated with the synergistic combination of C-PEI@OPDA deposition, ZW growth, and MPS grafting, the IFSS of the composites reached its maximum value. The above results indicate that the synergistic effect of multiple interfacial interaction mechanisms can effectively improve the interfacial strength between carbon fiber and PI resin.

**Figure 13:**
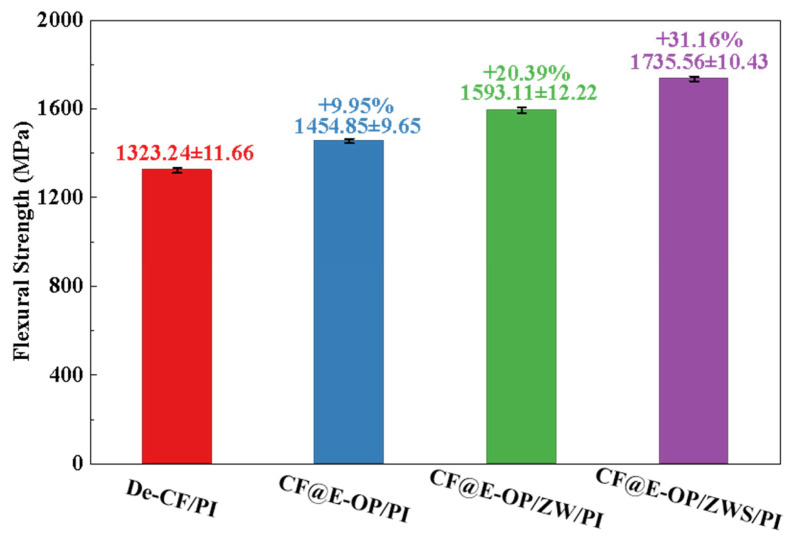
Flexural strength of CF/PI before and after modification, each group was tested five times, and data are presented as mean ± SD (n = 5).

Flexural strength reflects the overall load-bearing capacity of materials under bending loads. As shown in Figure 13, compared with De-CF/PI, the flexural strength of CF@E-OP/PI increased by 9.95%. This improvement benefits from the abundant hydrogen bonding interactions and dense resin crosslinked network in the interfacial layer, both of which constructed a stable interfacial layer. When the material is subjected to bending loads, external stress can be effectively transferred from the resin matrix through the interfacial layer to the carbon fiber, thereby improving the flexural strength of the composites. Compared with De-CF/PI, the flexural strength of CF@E-OP/ZW/PI increased by 20.39%, which is attributed to the micro-nano scale rough structures constructed by ZW on the carbon fiber surface. During the composite molding process, the molten resin penetrated into the ZW gaps and formed mechanical interlocking with the carbon fiber after curing. This strong anchoring effect can improve the stress transfer efficiency from resin to carbon fiber. Furthermore, during the process of the composite bearing bending loads, ZW can impede and deflect cracks at the interface, which increases the energy required for interfacial failure. On the basis of the aforementioned modifications, MPS was further grafted onto the carbon fiber surface. Compared with De-CF/PI, the flexural strength of the prepared CF@E-OP/ZWS/PI increased by 31.16%. This is attributed to the fact that the introduction of covalent bonds further improved the interfacial bonding strength between carbon fiber and PI, thereby enhancing the overall load-bearing capacity of the composites.

**Figure 14:**
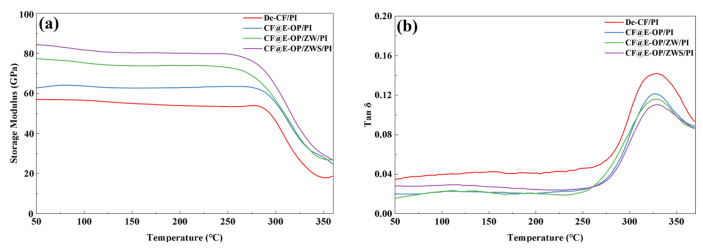
(**a**) Storage modulus and (**b**) loss factor of De-CF/PI, CF@E-OP/PI, CF@E-OP/ZW/PI and CF@E-OP/ZWS/PI.

The interfacial bonding strength between carbon fiber and resin matrix is positively correlated with the storage modulus of composites and negatively correlated with the loss factor (tan δ). As shown in Figure 14a, the storage moduli of CF@E-OP/PI, CF@E-OP/ZW/PI, and CF@E-OP/ZWS/PI were 62.72 GPa, 77.34 GPa, and 84.23 GPa, respectively, showing a gradually increasing trend. Compared with De-CF/PI (56.97 GPa), they increased by 10.09%, 35.76%, and 47.85%, respectively. For CF@E-OP/PI, the C-PEI@OPDA coating contains abundant –NH_2_ groups, and the active hydrogen in these –NH_2_ can promote the crosslinking and curing reactions of norbornene end groups in PI resin molecular chains, thereby forming a denser resin crosslinked network structure in the interfacial region and improving the stiffness of the interfacial layer. Meanwhile, the polar groups (such as –OH and –NH_2_) in the C-PEI@OPDA coating form abundant hydrogen bonds with PI resin, thereby enhancing the compatibility between fiber and matrix, making the interfacial bonding tighter and improving the stress transfer efficiency. Therefore, the storage modulus was improved compared with De-CF/PI. For CF@E-OP/ZW/PI, while retaining the above interfacial enhancement mechanism of the C-PEI@OPDA coating, the in situ grown ZW significantly increased the surface roughness of carbon fiber. During the composite molding process, molten PI resin penetrated into the gaps between nanowires and formed mechanical interlocking structures with the fiber after curing. This physical interlocking further improved the interfacial bonding strength and stress transfer efficiency, resulting in a further increase in storage modulus. For CF@E-OP/ZWS/PI, on the basis of the aforementioned dual interfacial enhancement mechanisms, the –SH groups in the grafted MPS molecules can form covalent bonds with the norbornene groups in PI resin molecular chains through thiol-ene click reactions. Compared with hydrogen bonds, covalent bonds have higher bond energy and provide more robust interfacial bonding. Therefore, CF@E-OP/ZWS/PI exhibited the highest storage modulus. As shown in Figure 14b, compared with De-CF/PI, the tan δ peak values of CF@E-OP/PI, CF@E-OP/ZW/PI, and CF@E-OP/ZWS/PI decreased successively. The reasons can be explained from the perspective of interfacial molecular motion and energy dissipation mechanisms. For CF@E-OP/PI, the –NH_2_ in the C-PEI@OPDA coating promoted the crosslinking and curing of PI resin in the interfacial region, and the formed dense crosslinked network restricted the mobility of resin molecular chains at the interface. Additionally, the hydrogen bonds formed between polar groups in the coating and PI resin further constrained the relaxation motion of molecular chain segments. The restricted molecular chain motion in the interfacial region led to reduced energy dissipation, and therefore the tan δ value decreased compared with De-CF/PI. For CF@E-OP/ZW/PI, on the basis of the above molecular chain constraint mechanism, the mechanical interlocking structure formed between ZW and PI resin effectively prevented relative slippage between fiber and matrix under dynamic loading, reducing frictional energy dissipation caused by slippage at the interface. The synergistic effect of mechanical interlocking and molecular chain constraint further reduced energy dissipation, and the tan δ value decreased accordingly. For CF@E-OP/ZWS/PI, the covalent bonds formed between –SH in MPS and PI resin firmly connected the fiber and matrix together. The energy required to break covalent bonds is much higher than that needed to overcome hydrogen bonds or mechanical interlocking. Therefore, under dynamic loading, molecular chain disentanglement and slippage were less likely to occur at the interface, resulting in minimal energy dissipation. Under the synergistic effect of the triple mechanisms of hydrogen bonding, mechanical interlocking, and chemical bonding, the tan δ value of CF@E-OP/ZWS/PI reached the lowest, indicating the best interfacial bonding quality.

**Figure 15:**
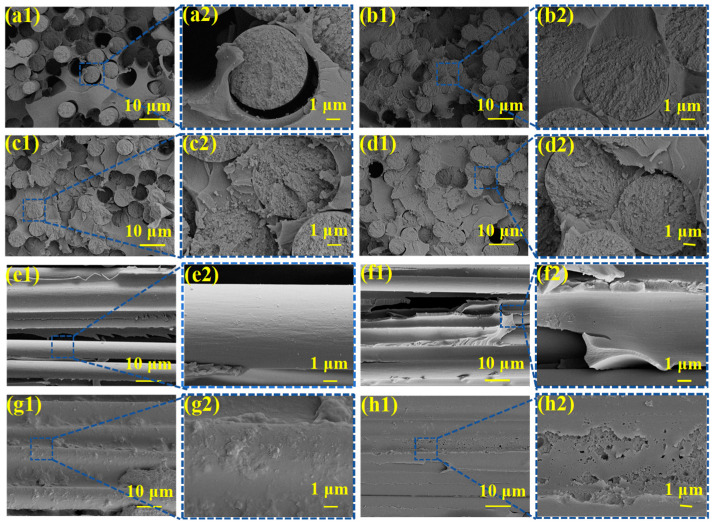
SEM of radial fracture surface: (**a1**,**a2**) De-CF/PI, (**b1**,**b2**) CF@E-OP/PI, (**c1**,**c2**) CF@E-OP/ZW/PI, and (**d1**,**d2**) CF@E-OP/ZWS/PI; SEM of latitudinal fracture surface: (**e1**,**e2**) De-CF/PI, (**f1**,**f2**) CF@E-OP/PI, (**g1**,**g2**) CF@E-OP/ZW/PI, and (**h1**,**h2**) CF@E-OP/ZWS/PI.

SEM was used to observe the radial and latitudinal fracture surface morphologies of De-CF/PI, CF@E-OP/PI, CF@E-OP/ZW/PI, and CF@E-OP/ZWS/PI to investigate their failure and fracture behaviors under external stress. For De-CF/PI, the radial fracture surface (as shown in Figure 15a1,a2) exhibited numerous holes and gaps, which can be attributed to debonding between fiber and resin matrix, leading to fiber pull-out. The corresponding latitudinal fracture surface (as shown in Figure 15e1,e2) was relatively smooth, indicating that the failure mode of this composite was dominated by interfacial failure. For CF@E-OP/PI, compared with De-CF/PI, the number of holes caused by fiber pull-out on the fracture surface decreased, and most carbon fibers were tightly wrapped by resin (as shown in Figure 15b1,b2). This indicates that after C-PEI@OPDA treatment, the interaction between carbon fiber and resin was enhanced, thereby improving the interfacial adhesion properties of the composites. The corresponding latitudinal fracture surface (as shown in Figure 15f1,f2) showed residual resin adhered to the fiber surface, but some smooth carbon fibers were still visible, indicating that the failure mode of this composite was a mixed mode of interfacial failure and resin fracture. For CF@E-OP/ZW/PI, the radial fracture surface was relatively flat, and fiber pull-out was further reduced (as shown in Figure 15c1,c2). This indicates that after growing ZW on the C-PEI@OPDA coating on the carbon fiber surface, the interfacial strength was further improved through mechanical interlocking. The corresponding latitudinal fracture surface (as shown in Figure 15g1,g2) showed that the fiber surface was completely covered by resin, indicating that cracks propagated within the resin rather than along the interface, which further confirmed the interfacial stability of this composite, and the failure mode was resin fracture. For CF@E-OP/ZWS/PI, it can be observed that the radial fracture surface of the composite exhibited almost no fiber pull-out (as shown in Figure 15d1,d2), the fiber surface was covered with a substantial resin layer, and the gaps between fibers were filled with resin. Crucially, on its latitudinal fracture surface (as shown in Figure 15h1,h2), a thick and highly rough layer of deformed resin was firmly adhered to the fiber, presenting obvious tearing characteristics. These indicate that the chemical bridge constructed between fiber and resin through MPS further improved the interfacial strength of the composites, thereby effectively inhibiting fiber debonding and crack propagation at the interface.

**Figure 16:**
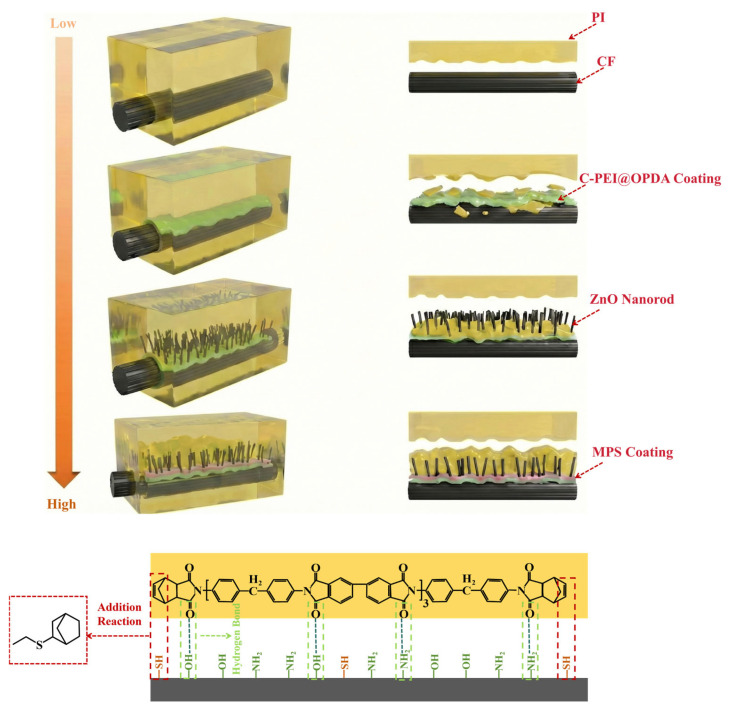
Interface failure mechanism of De-CF/PI, CF@E-OP/PI, CF@E-OP/ZW/PI and CF@E-OP/ZWS/PI.

Figure 16 illustrates the interfacial failure mechanisms of CF/PI before and after modification. As shown in the figure, since the desized carbon fiber surface exhibited chemical inertness, there was a lack of interaction force between the fiber and PI resin matrix, resulting in poor compatibility and easy formation of defects at the interface. Therefore, De-CF/PI failed due to interfacial debonding under relatively small stress. After C-PEI@OPDA modification, the carbon fiber surface was enriched with polar functional groups such as –OH and –NH_2_, which increased the hydrogen bonding between CF@E-OP and PI resin while improving the compatibility between carbon fiber and resin. Under this effect, the fiber and resin adhered more tightly at the interface with enhanced adhesion. Meanwhile, the active hydrogen in –NH_2_ can catalyze the addition reactions between olefins in PI molecular chains, thereby forming a denser resin crosslinked network structure in the interfacial region. Under the synergistic effect of the above two mechanisms, the interfacial quality was improved, and therefore greater external stress was required to destroy the interfacial bonding between fiber and resin in CF@E-OP/PI. When ZW was further grown in situ on the C-PEI@OPDA coating, while retaining the interfacial improvement effect of polar groups, mechanical interlocking was also introduced, thereby further improving the interfacial stability between fiber and resin phases. After grafting MPS containing –SH onto CF@E-OP/ZW, chemical bonds can be formed between CF@E-OP/ZWS and PI resin. While retaining the aforementioned interfacial enhancement mechanisms, a chemical “bridge” was constructed. Compared with hydrogen bonding and other interactions, breaking chemical bonds requires greater energy. Therefore, the failure mode of CF@E-OP/ZWS/PI was dominated by resin tearing rather than cracking at the interface.

## 4. Conclusions

This study systematically explored a multi-level interface modification strategy: first constructing a C-PEI@OPDA coating on the carbon fiber surface, then in situ growing ZW using the coating as a growth template, and finally grafting MPS onto the ZW surface. The C-PEI@OPDA coating, rich in –NH_2_ and –OH groups, not only enhanced the adhesion between the coating and carbon fibers but also adsorbed Zn^2+^ via coordination interactions to provide nucleation sites for ZW growth. Meanwhile, the active hydrogen in the coating promoted the crosslinking and curing reactions of PI resin, increasing the resin crosslinking density in the interfacial region. The vertically aligned ZW significantly increased the surface roughness of carbon fibers, and their abundant surface –OH groups provided secondary reaction sites for MPS grafting, successfully constructing a C-PEI@OPDA/ZW/MPS multi-level composite functional layer on the carbon fiber surface. The prepared CF@E-OP/ZWS composites achieved ILSS, IFSS, and flexural strength values of 75.15 MPa, 102.93 MPa, and 1735.56 MPa, representing improvements of 39.09%, 48.79%, and 31.16%, respectively, compared to untreated carbon fiber composites. The interfacial enhancement mechanism primarily manifests as the synergistic effects of multiple mechanisms: the C-PEI@OPDA coating enhanced fiber-resin compatibility through hydrogen bonding and increased the interfacial resin crosslinking density; the ZW enhanced the mechanical interlocking effect at the interface; and the –SH groups in MPS molecules formed chemical bonds with norbornene groups in PI resin through thiol-ene click reactions. These multiple effects mutually promoted and synergistically enhanced the interfacial properties of the composites. This research provides a theoretical foundation and practical reference for the interface design and optimization of high-performance CF/PI.

## Figures and Tables

**Table 1:** Growth parameters of ZnO nanorods.

Growth solution volume	300 mL
Temperature	90 °C
Time	10 h

**Table 2:** Grafting process parameters of MPS.

Solvent	Ethanol/water (1:1, *v*/*v*), 500 mL
pH	5
MPS concentration	0.5 wt%
Hydrolysis condition	Room temperature, 6 h
Grafting temperature	70 °C
Grafting time	2 h
Drying	120 °C, 2 h

**Table 3:** Summary of sample designations, surface modification.

Sample Name	Treatment	Purpose
De-CF	Commercial carbon fibers were desized by heat treatment at 380 °C for 2 h in an air atmosphere	Remove commercial sizing agent and expose clean fiber surface for subsequent activation
CFO	Liquid-phase oxidation treatment was applied to the De-CF surface using hydrogen peroxide solution	Introduce oxygen-containing functional groups (–OH, –COOH) to enhance surface chemical activity
CF@E-OP	Coating with C-PEI@OPDA polymer coating on CFO	Provide high-density polar groups (–OH, –NH_2_) to promote interfacial curing of polyimide resin
CF@OP	Coating with PDA polymer coating on CFO	As reference group
CF@ZW	ZnO nanorod samples grown on CFO	As reference group
CF@E-OP/ZW	In situ ZnO nanorod growth on CF@E-OP surface	Increase roughness; enhance interlocking
CF@E-OP/ZWS	MPS silane coupling agent was grafted onto the surface of CF@E-OP/ZW	Thiol groups form covalent bonds with norbornene groups; consolidate adhesion

**Table 4:** Composition of surface elements of carbon fiber.

Sample	Element Contents (%)					
	C	O	N	Zn	Si	S
CFO	73.80	26.2	-	-	-	-
CF@OP	75.41	18.64	5.95	-	-	-
CF@E-OP	74.76	17.74	7.50	-	-	-
CF@E-OP/ZW	65.11	27.08	5.12	2.69		
CF@E-OP/ZWS	53.27	27.27	4.35	2.22	11.76	1.13

**Table 5:** Chemical bond composition on carbon fiber surface.

Chemical Group	Peak Position	Functional Group Content (%)				
		CFO	CF@OP	CF@E-OP	CF@E-OP/ZW	CF@E-OP/ZWS
C–C/C=C	284.8 eV	75.81	55.55	57.80	-	-
C–NH_2_	285.5 eV	-	16.67	20.23	-	-
C–OH	286.2 eV	17.39	15.56	10.40	-	-
C=N	287.1 eV	-	5.00	7.51	-	-
C=O	288.7 eV	6.80	7.22	4.06	-	-
Zn–O	531.5 eV	-	-	-	52.63	59.52
O_V_	532.1 eV	-	-	-	30.00	26.20
–OH	532.7 eV	-	-	-	17.37	14.28

## Data Availability

The original contributions presented in this study are included in the article/Supplementary Material. Further inquiries can be directed to the corresponding author.

## Supplementary Materials

The following supporting information can be downloaded at: https://www.mdpi.com/article/10.3390/ma19050960/s1, Figure S1: (a) Preparation schematic of CF/PI composite laminates; (b) ILSS samples and (c) ILSS test schematic diagram; Figure S2: Process picture of IFSS test; Figure S3: Schematic of carbon fiber monofilament tensile sample preparation; Figure S4: Sample preparation and test method for water contact angle.
