# Supplementary material for: Mussel-Inspired Adhesive Layer Supporting ZnO Nanorod Arrays Combined with Thiol-Ene Click Reaction for Constructing Multi-Level Carbon Fiber/Norbornene-Polyimide Interfaces
Source: Materials (Basel). 2026 Mar 2;19(5):960. doi: 10.3390/ma19050960 (PMC12985497; doi:10.3390/ma19050960)
Supplement: Supplementary file 1 [file materials-19-00960-s001.zip › materials-4141290-supplementary.pdf]

materials

MDPI

Article

Mussel-Inspired Adhesive Layer Supporting ZnO Nanorod Arrays Combined with Thiol-Ene Click Reaction for Constructing Multi-Level Carbon Fiber/Norbornene-Polyimide Interfaces

Guoqiang Kong [1], Jianshun Feng [2], Meng Shao [1], Qiubing Yu [1], Zhenyu Liu [1], Kang Wang [1], Guang Yu [1], Xiang Zhao [1], Yan Huo [1], Xiaolei Guo [1], Qifen Wang [1], Zhe Sun [3], Haixiao Huang [4], Junwei Yu [2], Dayong Li [1] and Bo Zhu [2,*]

[1] Shandong Institute of Nonmetallic Materials, Jinan 250031, China; kongguoqiang2010@163.com (G.K.); sm41603057@163.com (M.S.); ayesdu2024@163.com (Q.Y.); yunszc53@163.com (Z.L.); wangk53@126.com (K.W.); gyyg2024@163.com (G.Y.); zhaoxiang9495@163.com (X.Z.); hy2024082026@163.com (Y.H.); gxl202408@163.com (X.G.); wangqifen0224@163.com (Q.W.); 15965639188@163.com (D.L.)

[2] Key Laboratory for Liquid Solid Structural Evolution and Processing of Materials, Ministry of Education, School of Materials Science and Engineering, Shandong University, Jinan 250061, China; fjs202408@163.com (J.F.); yujunwei@sdu.edu.cn (J.Y.)

[3] School of Electronic Engineering, Xidian University, Xi'an 710071, China; sz2024080123@163.com

[4] Shandong University Institute for Advanced Studies in Education, Jinan 250100, China; huanghx912@sdu.edu.cn

* Correspondence: zhubo@sdu.edu.cn

Academic Editor: Jorge R Oliva

Received: 23 January 2026
Revised: 22 February 2026
Accepted: 24 February 2026
Published: date

Copyright: © 2026 by the authors. Submitted for possible open access publication under the terms and conditions of the Creative Commons Attribution (CC BY) license.

Experimental Materials

The PAN-based carbon fiber (T700, 12 K, d = 7 μm) was sourced from Toray Industries, Inc. (Tokyo, Japan). The norbornene-terminated polyimide precursor solution (PI-NA, 50 wt%) was purchased from the Institute of Chemistry, Chinese Academy of Sciences (Beijing, China). 3-Mercaptopropyltrimethoxysilane (MPS, 97%), zinc acetate dihydrate ($Zn(CH_3COO)_2{\cdot}2H_2O$, AR, 99%), zinc nitrate hexahydrate ($Zn(NO_3)_2{\cdot}6H_2O$, AR, 99%), and hexamethylenetetramine (HMTA, AR, 99%) were supplied by Macklin Biochemical Co., Ltd. (Shanghai, China). Dopamine hydrochloride (DA·HCl, 98%) and tris(hydroxymethyl)aminomethane (Tris, 99%) were obtained from Aladdin Reagent Co., Ltd. (Shanghai, China). Polyethyleneimine (PEI, M.W. ~3000, 99%), absolute ethanol (AR, 99.7%), sodium hydroxide (NaOH, AR, 96%), hydrochloric acid (HCl, AR, 36–38%), and 30% hydrogen peroxide (H_2O_2, AR) were purchased from Sinopharm Chemical Reagent Co., Ltd. (Shanghai, China). Deionized water was prepared in house using a laboratory water purification system. All reagents were used as received without further purification.

Preparation of Prepregs

The preparation process flow of carbon fiber composites is shown in Figure S1a. For prepreg preparation, four types of carbon fibers (De-CF, CF@E-OP, CF@E-OP/ZW and CF@E-OP/ZWS) were uniformly coated with PI-NA solution, maintaining a mass ratio of fiber to resin at 6:4. The coated carbon fiber bundles were dried at 40 °C for 24 hours in an

Electric Thermostatic Blast Drying Oven (01-E, Beijing Yongguangming Medical Instrument Factory, Beijing, China) to produce prepregs.

Compression Molding of CF/PI Composites

Twenty-four bundles of prepared prepregs were arranged systematically in a mold for hot-pressing treatment using a Hot Pressing Machine (QLLHY-50T, Xiamen Qunlong Instrument Co., Ltd., Fujian, China). The hot-pressing process included four stages: holding at 120 °C for 100 minutes, at 200°C for 90 minutes, at 280 °C for 60 minutes (pressure was applied during this stage), and finally at 320 °C for 240 minutes. The composites prepared with De-CF, CF@E-OP, CF@E-OP/ZW, and CF@E-OP/ZWS as reinforcements were designated as De-CF/PI, CF@E-OP/PI, CF@E-OP/ZW/PI, and CF@E-OP/ZWS/PI, respectively.

Experimental Characterization

ILSS

The interlaminar shear strength (ILSS) of the composites was determined through a short beam test using Universal Testing Equipment (CMT4204, MTS-SANS, Guangdong, China) in accordance with the JC/T 773-2010 standard. The tests were conducted under controlled environmental conditions at 25 °C and 50% relative humidity. The specimens were positioned on supports with a 10 mm span, and a central bending load was applied at a rate of 1 mm/min, as illustrated in Figure S1(b, c). The test concluded when either the specimen failed or the maximum load was reached, at which point the failure mode and peak load were recorded. Each sample was tested at least five times, and the average value was calculated. The formula used for calculating ILSS is provided below:

$$ILSS = 3P/4bh \quad (1)$$

where ILSS is the short beam strength (MPa), P is the failure load (N), b is the specimen width (mm), and h is the specimen thickness (mm).

IFSS

The interfacial shear strength (IFSS) between the modified carbon fiber and resin was measured using a micro-bond test (model HM410, Tokyo, Japan). The sample preparation and testing procedures for IFSS are illustrated in Figure S2. The carbon fiber monofilament was secured onto an iron frame, and a 20 wt% PI-NA precursor solution was prepared. Then, the PI-NA precursor solution was brushed onto the carbon fiber monofilament, thereby forming droplets on the carbon fiber surface due to surface tension. Next, the iron frame holding the carbon fiber monofilament was placed in an oven and heated for 1 hour at 120 °C, 200 °C, 280 °C, and 320 °C, respectively, to promote the conversion of PI-NA precursor to PI-NA on the surface of the carbon fiber and to evaporate the solvent.

The heat-treated carbon fiber monofilament was removed from the iron frame, attached to a suitable paper frame, and mounted on the interfacial performance testing device. During the test, a symmetrical droplet with a size of 40-60 μm was selected, and the pulling rate was set to 1 μm/s. Each sample was tested at least five times, and the average value was calculated. The IFSS test result was determined by recording the maximum tensile load during the microdroplet debonding process. The calculation formula is as follows:

$$IFSS = F/\pi dl \quad (2)$$

where F is the maximum load (N) when the fiber is pulled out, d is the diameter of the fiber filament (m), and L is the length of the resin droplet (m).

Flexural strength

According to GB/T 3356-2014, the carbon fiber composite laminates were cut into specimens for flexural strength testing, with the following dimensions: specimen width w = 12.5 mm, specimen thickness h = 1.9 mm, span-to-thickness ratio L/h = 32, and specimen length L_0 = 75 mm (where L represents the span). The test was terminated upon specimen failure. The testing conditions were as follows: loading rate of 2 mm/min, with a minimum of five specimens tested for each sample. The flexural strength was calculated according to the following equation:

$$\sigma_f = 3PL/(2wh^2) \quad (3)$$

In this equation, σ_f represents the flexural strength (MPa), and P represents the maximum load sustained by the specimen (N).

DMA

The dynamic mechanical properties of the carbon fiber reinforced PI composites were characterized using a Dynamic Mechanical Analyzer (DMA Q800, TA Instruments, New Castle, DE, USA) in accordance with ASTM D7028-07. The test was conducted in three-point bending mode with specimen dimensions of 55 mm × 8 mm × 2 mm and a span of 50 mm. The testing parameters were as follows: frequency of 1 Hz, amplitude of 15 μm, heating rate of 3 °C/min, and temperature range from 50 to 365 °C. All tests were performed under a nitrogen atmosphere. The storage modulus (E') and loss factor (tan δ) as functions of temperature were obtained to evaluate the interfacial bonding quality of the composites.

Single-Fiber Tensile Testing

The tensile strength of the carbon fiber was measured using a Fiber Tensiometer (XQ-1C, XinXian, China). The stretching standard for carbon fiber monofilament is GB/T 31290-2022. The process of preparing single carbon fiber tensile specimens is shown in Figure S3. The ends of the carbon fiber monofilament were fixed onto a piece of paper, with a testing span of 20 mm. The paper was then secured on the testing device, and the test was conducted at a tensile rate of 2 mm/min. For each set of samples, at least 35 fibers were tested, and the average tensile strength was calculated. The formula for determining tensile strength is as follows:

$$\sigma = 4F/\pi d^2 \quad (4)$$

where σ is the single carbon fiber's tensile strength (Pa), F is the maximum breaking load (N), and d is the single carbon fiber's diameter (m). The results were statistically analyzed using the Weibull distribution model.

FTIR

The evolution of functional groups during the preparation of C-PEI@OPDA was analyzed using Fourier Transform Infrared Spectroscopy (FTIR, VERTEX-70, Bruker, Maryland, USA). The FTIR analysis was performed with the KBr pellet technique, scanning a wavenumber range of 400 to 4000 cm^{-1}.

SEM Testing

Field emission scanning electron microscopy (FE-SEM, model SU-70, JEOL, Ibaraki, Japan) was employed to analyze the surface morphology of the carbon fiber and the fracture surface characteristics of the composite material. A test voltage of 15 kV was applied

during SEM analysis, and prior to testing, samples were subjected to a gold sputter coating for 30 s.

XPS Testing

X-ray photoelectron spectroscopy (XPS, AXIS ULTRA, Kratos Analytical, Manchester, UK) was employed to investigate the alterations in elemental composition and functional groups on the surface of carbon fibers. The X-ray emission was generated from a non-monochromatic ultra-high vacuum (UHV) source utilizing the Mg Kα line (12 kV, 200 W) as anode. Following an initial survey scan, a high-resolution scan was conducted at a pass energy of 10 eV to elucidate the chemical state of the sample surface, encompassing both chemical composition and valence states of elements. The excitation energy applied is 1253.6 eV. To mitigate charging effects during analysis, fiber-shaped samples were trimmed to appropriate dimensions and affixed onto the sample holder using copper tape.

Contact Angle and Surface Energy

Using a contact angle goniometer (JC2000D1, Kezhong, Shanghai, China), the contact angles of carbon fibers before and after modification were measured by the static sessile drop method. As shown in Figure S4, the carbon fiber tow was laid flat on a glass slide and fixed at both ends with adhesive tape. During the fixing process, a certain tension was applied to the carbon fiber tow to ensure tight contact between the fibers and the glass slide, with the fiber bundles arranged closely together. The prepared contact angle measurement sample was placed horizontally on the stage for testing, with a droplet volume of 10 μL. The tangent method was used to fit the droplet profile to calculate the contact angle value. The contact angle was measured 5 times for each sample, and the surface energy was calculated using the Owens-Wendt method

XRD

The phase composition of the modified carbon fiber samples was analyzed using an X-ray diffractometer (XRD, D8 Advance, Bruker, Germany). The XRD testing conditions were as follows: Ni filter, CuKα radiation source, accelerating voltage of 40 kV, current of 50 mA, scanning speed of 10 °/min, and scanning range of 10-90°.

Raman

The structural evolution of the carbon fiber surface after C-PEI@OPDA coating modification was investigated by Raman spectroscopy (LabRAM-HR800, HORIBA Jobin Yvon, France) with a 532 nm laser.

Ultraviolet Spectrum

The concentrations of molecular functional groups in the DA and DA/PEI solutions were determined using a UV-vis spectrophotometer (UV-2600, Shimadzu, Kyoto, Japan). The measurements were performed in quartz cuvettes within a scanning range of 300–600 nm.

https://doi.org/10.3390/xxxxx

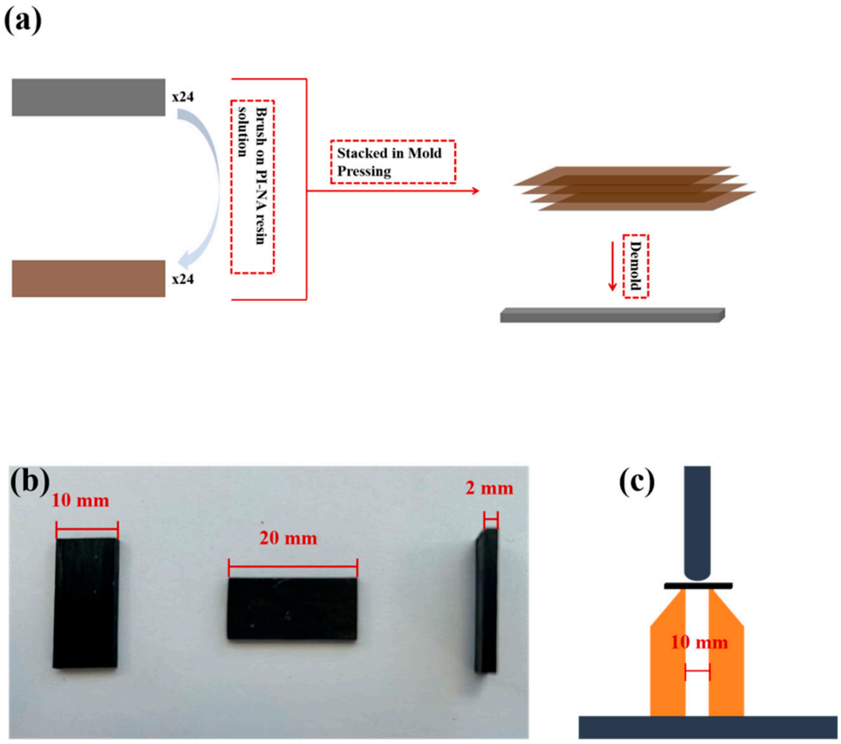

Figure S1. (a) Preparation schematic of CF/PI composite laminates; (b) ILSS samples and (c) ILSS test schematic diagram.

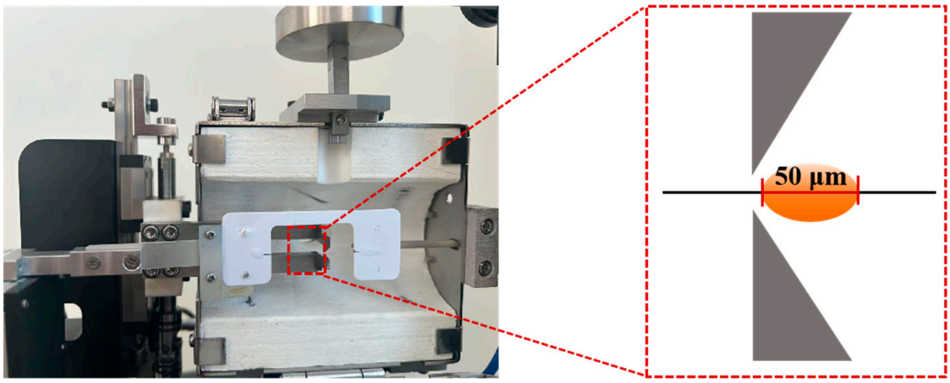

Figure S2. Process picture of IFSS test.

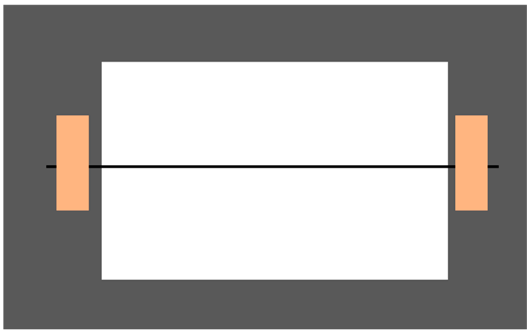

Figure S3. Schematic of carbon fiber monofilament tensile sample preparation.

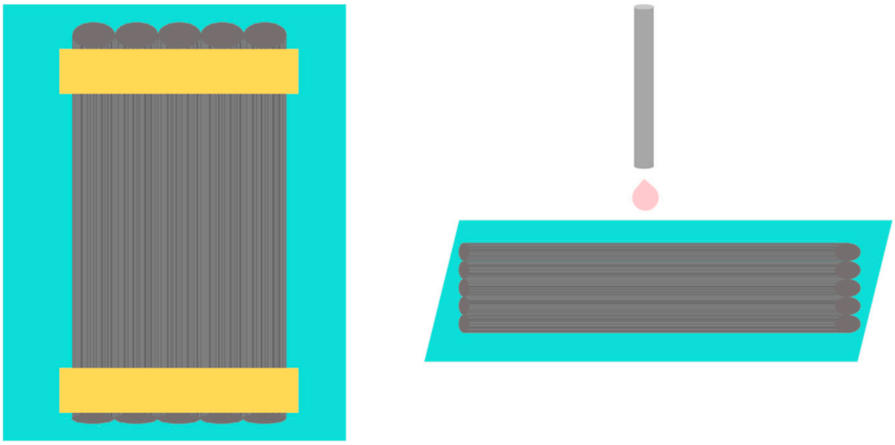

Figure S4. Sample preparation and test method for water contact angle.
